# Automated wearable cameras for improving recall of diet and time use in Uganda: a cross-sectional feasibility study

**DOI:** 10.1186/s12937-022-00828-3

**Published:** 2023-01-12

**Authors:** Andrea L. S. Bulungu, Luigi Palla, Joweria Nambooze, Jan Priebe, Lora Forsythe, Pamela Katic, Gwen Varley, Bernice D. Galinda, Nakimuli Sarah, Kate Wellard, Elaine L. Ferguson

**Affiliations:** 1 Department of Population Health, London School of Hygiene and Tropical Medicine, Keppel St, Bloomsbury, London, WC1E 7HT UK; 2 Department of Public Health and Infectious Diseases, University of Roma La Sapienza, 00185 Rome, Italy; 3 Department of Medical Statistics, London School of Hygiene and Tropical Medicine, London, WC1E 7HT UK; 4 School of Tropical Medicine and Global Health, University of Nagasaki, Nagasaki, 852-8102 Japan; 5 Africa Innovations Institute (AfrII), P.O Box 34981, Kampala, Uganda; 6 Department of Nutritional Sciences and Dietetics, Kyambogo University, Kyambogo, P.O. Box 1, Kampala, Uganda; 7 Natural Resources Institute (NRI), University of Greenwich, Chatham Maritime, Kent, ME4 4TB UK

**Keywords:** Wearable cameras, Feasibility, Acceptability, Uganda, Time use, Dietary assessment

## Abstract

**Background:**

Traditional recall approaches of data collection for assessing dietary intake and time use are prone to recall bias. Studies in high- and middle-income countries show that automated wearable cameras are a promising method for collecting objective health behavior data and may improve study participants’ recall of foods consumed and daily activities performed. This study aimed to evaluate the feasibility of using automated wearable cameras in rural Eastern Ugandan to collect dietary and time use data.

**Methods:**

Mothers of young children (*n* = 211) wore an automated wearable camera on 2 non-consecutive days while continuing their usual activities. The day after wearing the camera, participants’ dietary diversity and time use was assessed using an image-assisted recall. Their experiences of the method were assessed via a questionnaire.

**Results:**

Most study participants reported their experiences with the automated wearable camera and image-assisted recall to be good (36%) or very good (56%) and would participate in a similar study in the future (97%). None of the eight study withdrawals could be definitively attributed to the camera. Fifteen percent of data was lost due to device malfunction, and twelve percent of the images were "uncodable" due to insufficient lighting. Processing and analyzing the images were labor-intensive, time-consuming, and prone to human error. Half (53%) of participants had difficulty interpreting the images captured by the camera.

**Conclusions:**

Using an automated wearable camera in rural Eastern Uganda was feasible, although improvements are needed to overcome the challenges common to rural, low-income country contexts and reduce the burdens posed on both participants and researchers. To improve the quality of data obtained, future automated wearable camera-based image assisted recall studies should use a structured data format to reduce image coding time; electronically code the data in the field, as an output of the image review process, to eliminate ex post facto data entry; and, ideally, use computer-assisted personal interviews software to ensure completion and reduce errors. In-depth formative work in partnership with key local stakeholders (e.g., researchers from low-income countries, representatives from government and/or other institutional review boards, and community representatives and local leaders) is also needed to identify practical approaches to ensuring that the ethical rights of automated wearable camera study participants in low-income countries are adequately protected.

**Supplementary Information:**

The online version contains supplementary material available at 10.1186/s12937-022-00828-3.

## Background

Traditional recall approaches of data collection for assessing dietary intake and time use are prone to recall bias [1–3]. Prospective methods, which avoid recall bias, such as self-report diaries are not practicable in rural low-income country contexts due to low literacy, whereas direct observation is labor intensive. An alternative prospective approach is the use of automated wearable cameras. These devices are inexpensive technologies that prospectively and unobtrusively record activities as they are performed. Automated wearable cameras have been used to collect human behavior data in middle- and high-income countries, but their feasibility in rural, low-income country settings has not yet been determined.

**Fig. 1:**
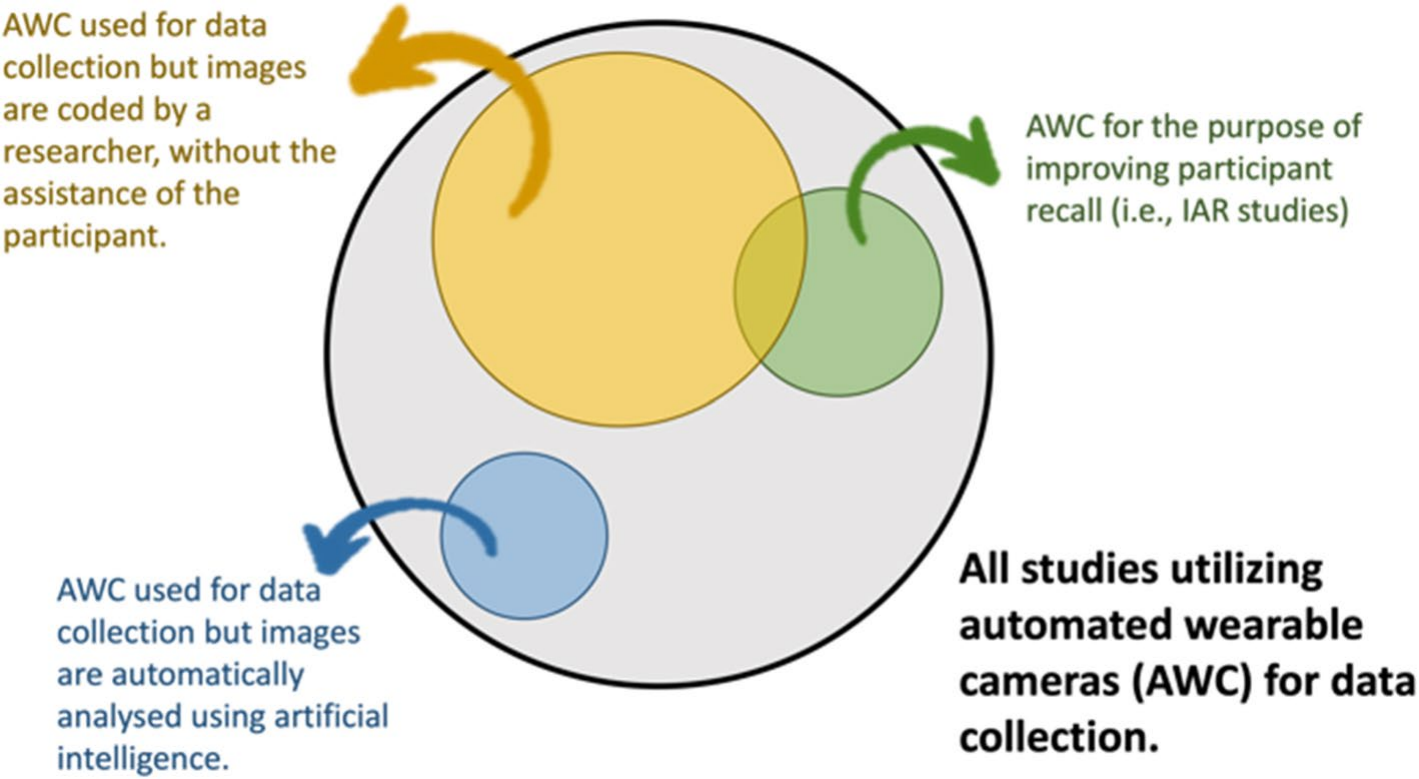
Illustration of the relationships between studies utilizing AWCs for data collection

Automated wearable cameras have been evaluated in middle- and high-income countries as a method for improving individuals’ recalls of dietary intakes (i.e., food and beverage consumption, eating episodes, and energy intakes) [4–12], the food environment (i.e., food and beverage marketing exposure, purchase, and consumption context) [6, 8], and time allocated to daily activities [13–15]. In studies using automated wearable cameras, the captured images have been coded by topical experts [16–40], artificial intelligence [41], or an enumerator with the assistance of the participant via an image-assisted recall [4, 6–9, 12–15, 42–47] (Fig. 1). In an image-assisted recall, photographs which have been taken either automatically via an automated wearable camera or by the participant themselves are used as an memory cue (i.e., recall trigger) to help respondents reconstruct key details from their previous day [14, 48–50]. Most image-assisted recall studies provide participants the opportunity to review and delete the images captured by the device privately, before being viewed by the researchers [51].

The "feasibility" of automated wearable cameras for collecting health data comprises an array of perceived and objective measures. Perceived measures include the emotional burden on study participants and the people they interact with; ease-of-use of the device; acceptability of image content captured by the wearable camera; and utility of the images captured by the wearable camera in aiding recall. Objective measures include participation refusal, non-compliance, and study withdrawal; device malfunction; observed or reported interactions regarding the camera with members of participants’ households and communities; image quality and fit for purpose; time and other resources required for image processing, coding, and analysis; and device cost. Feasibility issues can further be categorized by audience, i.e., from a participant, community, and/or researcher perspective.

### Feasibility from a participant perspective

In several studies, some participants found the automated wearable camera cumbersome to wear, especially during physical activity [4, 8, 14, 20, 29, 31, 45, 47, 52, 53]. Studies in which participants were responsible for operating the device (e.g., turning the automated wearable camera on and off at the start and end of the data collection day), commonly reported that participants forgot to wear or charge the device [23, 26, 29, 31, 35, 44], or had difficulty pressing the devices’ small buttons [5, 17, 52, 53]. Participants have also reported emotional discomfort due to wearing the device, especially in public [6, 12, 29, 34, 53]. Heightened awareness of an automated wearable camera may result in a reactive change in the study’s behavior of interest [4, 12, 16, 20, 29, 34]. In six studies participants reported having modified their behavior in reaction to being recorded.

Concerns about either wearing the camera or what it might capture may also negatively influence the rates of recruitment and completion. Response rates varied substantially across automated wearable camera-based studies (16% to 89% where reported) [4, 6, 20, 29, 35, 47]. Several of the studies, which explicitly investigated the impact of an automated wearable camera on response rates, attributed recruitment challenges to the device [6, 20, 29, 35, 53, 54]. Study withdrawal [8, 47] and non-compliance [14, 20, 31] have also been attributed to the use of automated wearable cameras.

For studies using the image-assisted recall method, participants across all age groups reported that viewing the images captured by the automated wearable camera helped them to recall pertinent details of the data collection period [4–8, 12, 14]. Participants reported that neither the length of time nor the process of reviewing their automated wearable camera-captured images (i.e., the image-assisted recall) was onerous [4, 14].

Among the 28 studies reporting on automated wearable camera feasibility from a participant perspective, only three were conducted outside of high-income country contexts [7, 40, 55]. The evidence from these three studies, which were conducted in middle-income countries, is sparse but consistent with the results already reported. There were no issues related to recruitment or retention, and neither the automated wearable camera nor the image-assisted recall was overly burdensome, however the battery life of the device was insufficient.

### Feasibility from a community perspective

Study participants reported removing or covering the automated wearable camera at school [8, 13, 14, 34], work [14], home [20, 31], and in public [45]. Three studies reported participants being approached about the automated wearable camera by members of the public, but they were not requested to remove it [44, 52, 53].

No studies outside of high-income country contexts have assessed the feasibility of using an automated wearable camera from the community perspective.

### Feasibility from a researcher perspective

Lost data due to device inoperability (e.g., insufficient battery life or another malfunction) is among the most commonly reported challenges to the use of automated wearable cameras as a research method [4–6, 9, 12, 16, 20, 30, 31, 34–36, 38, 41, 47, 56]. Reported data losses due to device inoperability, as a proportion of intended image capture, ranged from 11–50% [5, 6, 12, 35–37, 47, 57]. Most studies report that the images generated by the automated wearable camera are of sufficient quality to enable analysis for the intended purpose. However, several image quality issues are commonly reported across a variety of contexts, including sub-optimal camera angle and positioning, inadequate image capture frequency, and key events that occur off-camera; [4, 6, 8, 9, 12, 16, 18, 19, 21, 25, 31, 36, 38] dark images caused by low or artificial lighting or obscured lens; [5, 8, 12, 21, 23, 24, 28, 31–36, 44, 47, 52] and blurry or scrambled images [16, 21, 22, 27, 31–33, 36, 37]. Furthermore, automated wearable camera images have been reported to be unsuitable for detailed analyses, for specific research areas, such as determining specific items of clothing worn by children far away from the camera [21], or detecting low intensity activities (e.g., fidgeting or activities performed while sitting down) [19]. The proportion of automated wearable camera images reported to be "uncodable" ranged from 1–35% [17, 20–24, 27, 28, 30–34, 36, 39, 52].

The results were again sparse but consistent for the two studies conducted in middle-income countries with results related to automated wearable camera feasibility from a researcher perspective [40, 55]. In these studies, the cause of data losses was indeterminant and, although the image quality was acceptable, in one study the images captured were unfit for research purpose (determining the quantity of food consumed) [40].

The use of automated wearable cameras for research data collection aims to maximize reporting accuracy while minimizing participant burden. Part of this burden is shifted to the research team, and several studies highlight the heavy time burden required to manually code the automated wearable camera images for analysis [6, 14, 18, 24, 28, 29, 33, 34, 36, 38, 47], and its susceptibility to human error [24, 29]. Not all studies quantified the amount of time entailed, but where reported, the estimated time required to code automated wearable camera images range from approximately 1 to 2 h per participant day [7, 14, 17, 21, 23, 24, 27, 30, 31, 33–36, 38, 39]. Little information on other costs of automated wearable camera-based research is available. Only Kelly, et al. (2015) reported on the cost of the device (Autographer, £300 each), adding that it was "resource intensive" [14].

Although many studies acknowledged some feasibility limitations, especially for use in large-scale studies, nearly all concluded that automated wearable cameras are a promising method for collecting objective health behavior data in a free-living setting. Furthermore, despite the challenges described above, studies in high-income countries provide evidence that automated wearable cameras may help to improve study participants’ recall of foods consumed [9], and daily activities performed [14].

The available evidence for automated wearable camera feasibility, however, almost exclusively derives from studies conducted in high-income and upper-middle income countries. Key characteristics of rural women residents of low-income countries, such as literacy, exposure to technology and social norms, are quite different compared to any of the populations targeted in the automated wearable camera research published thus far. The research environment in rural low-income countries also poses different challenges including, for example, limited access to electricity for lighting the activity space or charging devices, and higher chance of device exposure to dirt or liquids, and lack of enumerators having pertinent skills. Feasibility needs to be explored in low-income countries, especially in rural contexts, given that the environmental conditions (which may affect device operability and image quality), social norms (which may affect acceptability by participants and the public), and familiarity with technological devices (which may affect ease-of-use) are all quite different than in high-income and/or upper-middle-income countries.

This study was therefore undertaken to assess the feasibility of using an automated wearable camera for data collection, in rural Eastern Uganda, on the dietary practices of women and young children and time-use patterns of women. The results can inform future automated wearable camera studies conducted in similar contexts.

## Methods

This study is presented per the Strengthen the Reporting of Observational Studies in Epidemiology (STROBE) protocol [58].

### Study design

This study was nested within a cross-sectional study of women with a child aged between 12 and 23 months inclusive (*n* = 211), to examine the impact of a labor-saving technology on women’s time for childcare, food preparation and dietary practices. It was conducted between January and February 2018 in Bugiri and Kamuli Districts, Eastern Region, Uganda. It validated the use of three methods of collecting data on dietary practices and women’s activities, which were the automated wearable camera-based image-assisted recall, interactive voice response collected via a mobile telephone, and 24-h recalls, using direct observation as the reference method. Only results related to the automated wearable camera-based image-assisted recall are reported here.

In our study, maternal and child dietary diversity and women’s time allocation were assessed via an image-assisted recall using photos captured the previous day with an automated wearable camera. The methods are described in detail elsewhere [3, 59]. However, in brief, for each respondent, dietary intake and time allocation data were prospectively collected using photographs automatically taken every 30 s by a wearable camera attached to the participant. The next day, using the photos captured by the automated wearable camera during the previous day, an enumerator first independently coded the images for foods / beverages consumed by the mother and child and activities performed by the mother. Then, the enumerator administered an image-assisted recall to the participant. On the day before data collection began, a structured socio-demographic questionnaire was administered, and anthropometric measurements were made. On the final day of data collection, a structured questionnaire was administered to assess participants’ perceptions of the automated wearable camera-based image-assisted recall method. Each participant wore the automated wearable camera for two non-consecutive days and completed two image-assisted recalls, which meant enumerators met participants on a total of four days to collect two days of data on dietary practices and women’s activities.

Ethical approval was obtained from the Uganda National Council for Science and Technology (UNCST) (A24ES), the London School of Hygiene & Tropical Medicine Observational Research Ethics Committee (Project ID: 1420), and the University of Greenwich Faculty of Engineering and Science Ethics Committee (Project ID: B0501). Community sensitization was done to ensure the study participants and other community members understood the study objectives and data collection methods. It included a review of key aspects of informed consent, demonstration of the devices used in the study (i.e., automated wearable camera, mobile phone, and GPS tracker), and a detailed description of the methods that would be used in the study, and time was allowed for questions. Following community sensitization, written informed consent (signature or thumb print) was obtained from all respondents who participated in the study.

### Participants and sampling

Twelve mother–child dyads were randomly selected from 22 purposefully selected villages in two districts of Eastern Region Uganda, as described elsewhere [3]. Mother–child dyads were excluded if the child was less than 12 months or greater than 23 months of age, was not yet eating solid foods on a regular basis, or was a multiple-birth child; the mother was unable to communicate in Lusoga, Luganda or English; either the mother or child had a severe disability; the mother was not the biological mother of the child; the mother was a co-wife with a selected mother; or either the mother or child was not available for the duration of the study. Participants were given a bar of soap, one kilogram of sugar, a half-liter of cooking oil and a t-shirt on the final day of the study. Also on the final day, they were given a photo of their family taken by a supervisor using a polaroid camera.

### Instruments and protocol

The enumerators administered two structured questionnaires to the respondent. The first questionnaire collected information on household socio-demographics and assets, and factors related to women’s empowerment. The second questionnaire, which was administered on the final day of data collection, collected information on household mobile phone access and ownership, and participants’ perceptions of their experiences with the automated wearable camera-based image-assisted recall and other data capture methods assessed in this study that are not reported here (i.e., direct observation, 24-h recall, and mobile phone-based interactive voice response). Specifically, participants were asked to rate the automated wearable camera-based image-assisted recall method using a 4-point Likert scale (very bad, bad, good, or very good). Participants were also asked to select their favorite and least favorite method among the four data capture methods assessed, and whether they would be willing to participate in an automated wearable camera-based image-assisted recall study again. Although not specifically requested, any comments provided by the participants in answering these questions were translated and transcribed by the enumerators. A brief "innovative methods’ questionnaire was also completed at the end of each data collection day to assess participants’ experiences wearing the automated wearable camera, including any technical issues or reactions from members of their households or communities. Each participant was also asked to reconfirm her consent to use the images captured by the automated wearable camera. No data on automated wearable camera acceptability among other members of the household or community were collected.

**Fig. 2:**
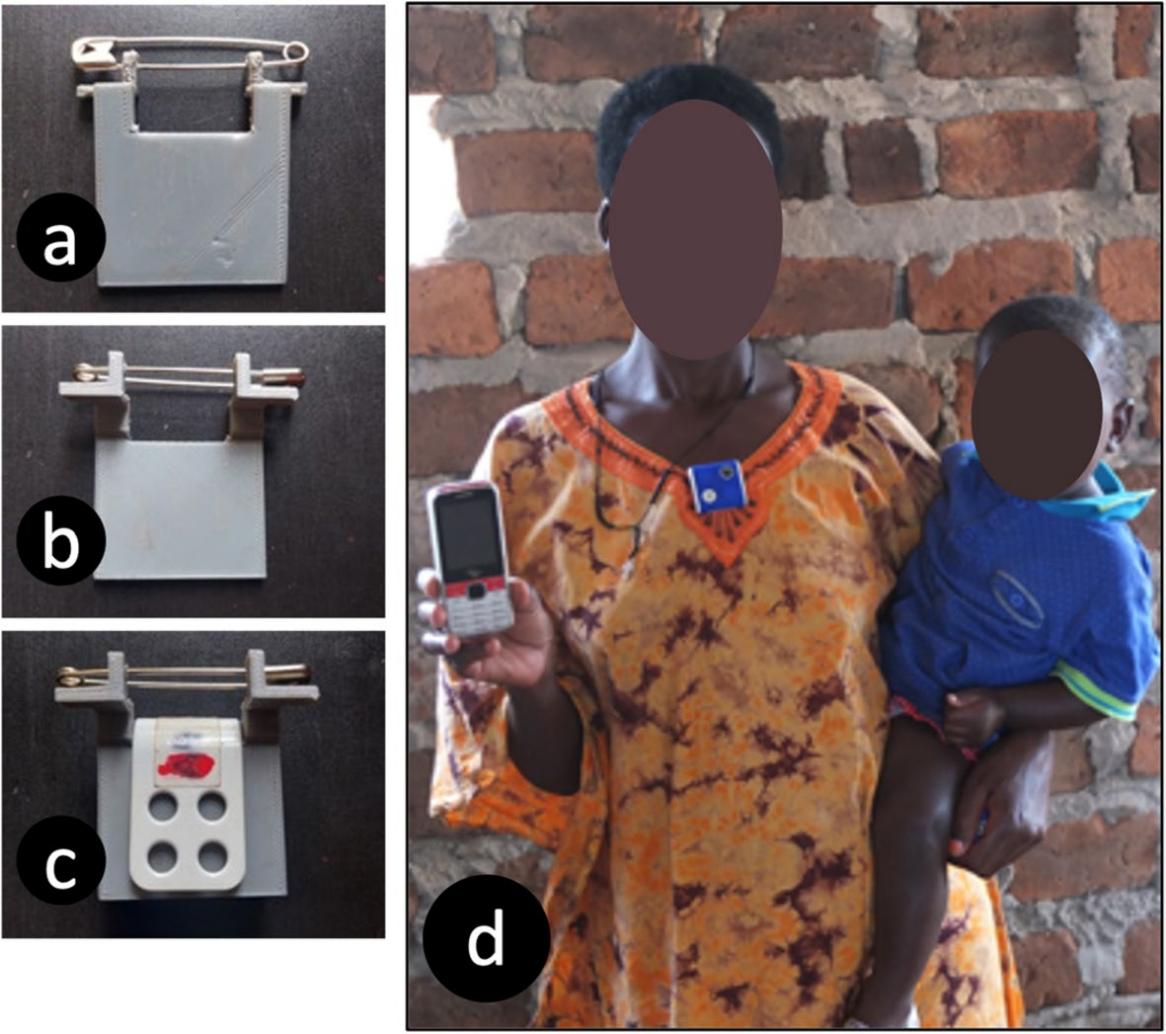
The AWC affixed via a bespoke clip to the neckline of participants' clothing

As described elsewhere [3], a small, lightweight, automated wearable camera (iON SnapCam Lite, dimensions 42 × 42 × 13 mm) was attached to a t-shirt worn by the respondent at approximately 06:00 and removed at approximately 21:00. Participants were instructed to wear the automated wearable camera while continuing their usual activities, and to cover or remove the camera as needed for privacy. A bespoke plastic clip using a safety pin was designed to keep the device firmly attached at the neckline of the t-shirt and well-positioned to minimize interference with clothing (Fig. 2). The wearable camera automatically recorded a picture every 30-s, storing all photos (approximately 1,500) on a micro-SD memory card and with the image number (e.g., 4) as the filename (e.g., SNAP0004.JPG). Examples of the photos obtained by device are provided in Supplementary Figure 1.

The automated wearable camera was turned on at the beginning of the day and turned off at the end of the day by the enumerator. The t-shirt was provided by the study and worn by the participant over her clothing, so that if the participant needed to remove the camera, she would remove the entire t-shirt rather than handling the device. For administrative purposes, at the start of each data collection day, the enumerator took a single picture of the participant, her child and a placard displaying her study ID using the designated function of the device. After attaching the automated wearable camera to the project-provided t-shirt, the enumerator reminded the participant of key points covered during sensitization, i.e., that at any time during the study she could remove or cover the device or request all images to be deleted; that the device was splash proof but could not withstand immersion in water; and to do exactly the activities she would have normally done. Upon picking up the automated wearable camera at the end of the data collection day, the enumerator completed the innovative methods questionnaire. In addition, the first author (ALSB) kept records of inoperable devices, and members of the data collection team monitored issues (e.g., negative rumors about the automated wearable cameras) that may have affected study participation or compliance.

Upon collection of the devices used by the study participants, ALSB saved a copy of the images recorded on each automated wearable camera memory card to a local drive, and assigned two participants’ memory cards (i.e., data for two participants) to an enumerator who had not been engaged in direct observation of the participant the previous day. The following day, the enumerator inserted the assigned memory card for the first participant into a tablet (16 GB Samsung with a 10″ screen, using Simple Gallery software for image display) to review the photos captured by the automated wearable camera. Using the image-assisted recall form, the enumerator annotated the foods she thought were consumed and the activities she thought were undertaken by the participant, and their corresponding image numbers, based on what she could see in the photos. Based on her interpretation, the enumerator demarcated the series of foods and activities for review later that day with the respondent. After completing the annotation for the first assigned participant, the enumerator completed the same steps for the second assigned participant.

Upon meeting with the participant, the enumerator oriented the mother to the photos captured by the automated wearable camera by viewing, on the tablet with the inserted memory card, five pre-selected images: a picture of the mother herself, a picture of her child, a picture of her home, a picture of her garden, and a picture where her own hand is visible while performing a task (e.g., while preparing or cooking food, digging, or using a mobile phone). The enumerator rated the participant’s ability to recognize the content of these five photos on a three-point scale: recognized, recognized with help, or failed to recognize. The enumerator then administered the image-assisted recall. During this interview, the enumerator used "verbal probing" [60, 61] to elicit from the participant additional relevant information about the activities performed, for example to elaborate on what she was doing, who she was with, where she was going and why, etc. The enumerator revised her original annotations of foods / beverages consumed and activities undertaken by the participant, as needed, based on the participant’s feedback.

The image-assisted recall protocol was adapted from one described by Kelly et al. (2015) [14]. The protocol followed ethical guidelines for automated wearable camera research to ensure privacy of the participants was maintained [51]. All protocols were pilot tested and refined prior to the start of the study.

Enumerator training for all devices, protocols, and instruments took place over one week (December 18–22, 2017). The training comprised classroom training, role-play practice, and an assessment with individualized feedback. Training also included two days of field practice.

### Data analysis

In this study, feasibility was assessed using administrative data (non-compliance and withdrawal; camera malfunction; image quality; researcher time allocated to data coding and analysis); participants’ self-reported ratings of their experiences with the automated wearable camera and other methods used in the study; enumerators’ ratings of participants’ ability to interpret the images captured by the wearable camera; non-technical (e.g., fear of health or spiritual harm caused by the automated wearable camera) and/or technical (e.g., depleted battery) issues regarding the wearable camera reported by study participants or members of the data collection team; and requests by participant to delete wearable camera captured images.

Participants’ self-reported experiences with the automated wearable camera and method ratings were double entered via EpiData. Administrative and demographic data and participant image-assisted recall orientation ratings were entered via Excel. Information about the data processing and analysis of demographic, dietary diversity and time-use data has been previously published [3, 62].

Because the data were not normally distributed, the Mann–Whitney U test and Fisher’s Exact test were used to compare method ratings for participating households and households lost to the study. Data were analyzed using Stata/SE version 17. *P*-values less than 0.05 were considered significant for all tests.

## Results

### Characteristics of the sample

Overall, 211 women were recruited into the study. Among those recruited, twenty-seven participants were eliminated from analysis due to incomplete image-assisted recall data (*n* = 27), including eight participants who voluntarily withdrew from the study (*n* = 8) and two participants who were unavailable for the image-assisted recall due to a funeral (*n* = 1) or medical emergency (*n* = 1) (Supplementary Fig. 2). The remaining seventeen (*n* = 17) instances of lost data were due to administrative errors (e.g., inadequate number of tablets or other enumerator-caused image-assisted recall non-complete). No differences in demographic data were found between participants who were excluded from the analyses and those who were analyzed (Supplementary Table 1). The median household size was six members, and approximately one-fifth of participating households lived below $1.25/day. The mean age of participants was 26 years. Most participants were married and had attended primary school. Only half of participants were literate. Slightly more than half of the participants identified as Christian.

### Feasibility from a participant perspective

Most participants rated their experience of wearing the automated wearable camera and reviewing the photos the following day (image-assisted recall) as either good (36%) or very good (56%) (Supplementary Table 2). For over a quarter of participants (26%), the automated wearable camera-based image-assisted recall was their favorite of the four research methods assessed, which was significantly higher than the proportion who preferred the 24-h recall method (4%) (Supplementary Table 3a-d). These participants reported that they enjoyed looking at the photographs and that the photographs helped them remember key details.

For over a quarter (29%) of study participants, the automated wearable camera-based image-assisted recall was their least favorite of the four research methods assessed. For these participants, invasion of privacy, fear of the device and fear of others’ reaction to the device (emotional burden) were contributing factors. This result was not significantly different than the proportions who rated direct observation (24%), 24-hour recall (32%), or the mobile phone-based interactive voice response (15%) as their least favorite method (Supplementary Table 4a-d).

Nearly all (96%) participants reported that they would be willing to wear an automated wearable camera to record their food intake and daily activities in a future study. Furthermore, none of the eight study withdrawals can be definitively attributed the automated wearable camera. In three cases, the participant’s husband declined after she had initially consented. One participant withdrew after expressing frustration with one of the other data capture methods (i.e., mobile phone-based interactive voice response) assessed in this study ("I am tired, tired, tired of your things"). Two reported being called away to attend a burial and no rationale for withdrawal was provided by the remaining dropouts (*n* = 2). Most (*n* = 6) of the withdrawals occurred in one of the two study districts (Kamuli) which resulted in a higher withdrawal rate in this district than in Bugiri (i.e., 9% in Kamuli vs 1% in Bugiri).

Across all participants, including those who withdrew, none requested that their image data be entirely deleted either at the end of the data collection day or after viewing their photos during the image-assisted recall. Seven participants requested that a few specific photos be deleted. In two cases, the participants were aware that the camera had likely captured private activities (bathing children and using the latrine) and requested pictures to be deleted at the time of device pick-up. In three cases, the participant vaguely indicated they wanted "a few" images deleted but did not specify which images at the time of data collection or during the image-assisted recall. In the remaining two instances, participants requested specific images to be deleted after seeing them in the image-assisted recall.

### Feasibility from a researcher perspective

An additional 15% of data (*n* = 27) had an insufficient number of images captured due to inoperability of the automated wearable camera. The causes and/or nature of these automated wearable camera malfunctions were usually unclear. In only about a half (*n* = 16) of these cases were operability issues reported at the time of data collection (usually identified by either the failure of the device to display a blue light indicating a photo had been taken or to beep in response to button pressing) by either the participant or a member of the research team. In five instances the enumerator inadvertently recorded a video, which may have depleted the device battery or storage capacity. There were also instances (*n* = 15) where an operability issue was reported but, if a malfunction occurred, it did not substantially reduce the number of images captured. The number and proportion of inoperable automated wearable cameras increased over the course of the study (Supplementary Table 5). Only 6% of the study population in Bugiri was affected by inoperable devices versus 26% in Kamuli. Photos taken by the automated wearable camera before dawn, in the evening, or indoors were often too dark to determine foods or activities. Overall, about twelve percent of images captured by the automated wearable camera were too dark to interpret.

Approximately half (53%) of participants had difficulty interpreting at least one of the five pre-selected "orientation" images (i.e., a picture of the mother herself, a picture of her child, a picture of her home, a picture of her garden, and a picture where her own hand is visible while performing a task). Ease-of-recognition of two types of orientation photos improved between the first and second image-assisted recall - the photo of the participant's garden (from 93% to 97%) and the photo of her own hand while performing a task (from 89% to 96%) (Supplemental Table 6).

## Discussion

### Principal Results

This is the first study to investigate the feasibility of an automated wearable camera-based image-assisted recall for collecting maternal and child dietary diversity or women’s time-use data in a low-income country context. We assessed the feasibility of the automated wearable camera-based image-assisted recall method with mothers of young children in rural Eastern Region Uganda using administrative data and participant-reported perceptions. Results showed the collection of food / beverage intake and women’s time-use data, using an automated wearable camera, was feasible, although data loss was high.

There were no reports of physical discomfort due to the automated wearable camera. Unlike previous studies that have hung the automated wearable camera on a lanyard, which may swing and get in the way of daily activities, in this study a bespoke clip was used to securely fasten the automated wearable camera to a t-shirt that was large enough to fit over the participant’s clothing. Camera malfunction was minimized because participants in this study were not responsible for operating the device. Accordingly, there also were no instances of lost data due to participants forgetting to wear the automated wearable camera or failing to recharge it, nor were any usability issues reported. There were, however, eight instances of inadvertent video recording, possibly indicating a usability issue with the device when operated by the trained enumerator. These cases can lead to lost data because video recording uses more battery and storage space.

Based on the high participant retention rate and the participants’ end-of-study method ratings, the acceptability of the automated wearable camera among participants of this study was high. Although a fifth of participants deemed it their least favorite method among those assessed in this study, 92% still rated the automated wearable camera-based image-assisted recall method as good or very good and 97% were willing to participate in an automated wearable camera-based image-assisted recall study in the future.

Although eight participants withdrew from the study, none could be definitively attributed to the automated wearable camera. Three of the eight withdrawals occurred because the participant’s spouse refused to participate (after the participant had consented), which underscores the importance of careful sensitization of not only potential participants but also their families and their communities prior to recruitment. Careful monitoring (e.g., for rumors) during the period of data collection is also critical to address any concerns before they become a more widespread problem. This also underscores the importance of having a field team that speaks the local language and is familiar with the local culture, as well as strong engagement with and support of community leaders. Notably, a higher proportion of the participants who withdrew were from Kamuli than Bugiri, which may indicate that social acceptability can vary across small-scale geographies. In small close-knit communities such as the ones where this study was conducted, a single negative event (e.g., illness or death of a community member or family member) or rumors (e.g., that the automated wearable camera causes spiritual harm) can influence participation. Because the automated wearable camera-based image-assisted recall method requires two days of data collection—one day to wear the camera and the next day to review the images captured by the automated wearable camera—it is vulnerable to unanticipated absences. Future research to explore the influence of social networks on automated wearable camera study participation may be warranted.

Participants in this study were offered multiple opportunities to delete all or some of the images captured by the automated wearable camera. None of the participants requested that their data be entirely deleted and only seven participants requested that a few specific photos be deleted. This low level of deletion indicates that participants found the content of the automated wearable camera images acceptable, or that they did not feel comfortable asking the enumerators for the photos to be deleted. Among the seven studies reporting on the acceptability of the content captured by the automated wearable camera (all in high-income countries), six reported participants opting to delete images [20, 24, 30, 31, 45, 53]. Drawing comparisons between this study and previous studies is difficult because, for most image-assisted recall studies, participants are not obligated to report if or how many images they deleted. The ethical framework outlined by Kelly et al. for the use of automated wearable cameras in health behavior research [51] recommends that participants are provided time to review their images in private before being viewed by the research team, and to delete any images desired. This approach is impractical in a low-literacy population with limited exposure to digital technologies. This constraint and other recommendations of the Kelly framework warrant review by key local stakeholders, such as researchers from low-income countries, representatives from government and/or other institutional review boards, and community representatives and local leaders, to identify practical approaches to ensuring that the ethical rights of automated wearable camera study participants in low-income countries are adequately protected.

This study was not designed to assess acceptability from the community perspective. In the end-of-day innovative methods questionnaire, however, there were no reported requests from the public to remove the automated wearable camera, although one participant reportedly removed the device after being "threatened by others that she is being recruited for the Illuminati" (supervisor’s field notes). Future studies need to investigate acceptability from the community perspective given the substantial socio-cultural differences between high-income country contexts and rural low-income country contexts, and across different low-income countries.

Fifteen percent of data was lost due to automated wearable camera malfunction (*n* = 27), which is similar to reported data losses due to device malfunction in other studies [5, 6, 9, 12, 35–37, 47]. The relatively low rate of device malfunction may be because they were operated by the enumerators rather than the participants, and could be quickly replaced when an enumerator was present on the observation day. Indeed, the number instances of lost data due to inoperability was higher on days when the observer was not present at the home (*n* = 24 and *n* = 32 when the observer was and was not present, respectively) (Supplementary Table 7). Nevertheless, any amount of lost data is a waste of study and participant resources and may decrease the power of the study to detect the outcomes intended. Automated wearable camera operational issues increased over the course of the study, which suggests that wear and tear on the devices or the SD cards rather than inadequate battery charging were at fault. Back-up devices may need to be procured so that malfunctioning automated wearable cameras in the field can be replaced. However, it was not always evident when a device was inoperable until the end of the data collection day when the images were downloaded. Future studies will need to over-recruit participants in anticipation of higher-than-usual attenuation compared with the 24-h recall or direct observation methods.

Overall, the quality of images captured by the automated wearable camera was sufficient for the purposes of assessing dietary diversity and time use, however, twelve percent of the images were "uncodable" due to insufficient lighting (i.e., too dark to determine foods or activities). This result is within the range of uncodable images reported in previous studies [17, 20–24, 27, 28, 30–34, 36, 39, 52]. Due to long periods of indecipherable activities at the beginning and end of each day, however, the time use validation study analysis was reduced from 15 to 12 h. Constraints on the period of data collection limits the comparability of results obtained via automated wearable camera-based image-assisted recall versus other methods, for example, because of missing behaviors of interest that only occur in low-lit contexts, such as storytelling with children before bed or consumption of more nutritious dishes in the evening than during the day. Future studies are advised to explore unobtrusive options for improving ambient light when the participant is indoors or in the morning or evening when sunlight is low.

In addition, automated wearable camera photos captured during vigorous activity, for example while sweeping or riding a bicycle, were sometimes blurry, although for the purposes of this study interpretation was not hindered when viewed in context of the surrounding images. The image quality was sometimes inadequate to determine slight color variations that are important for dietary assessment, such as the difference between white and yellow sweet potatoes, which would be important in studies where the coding was performed by the researcher without the assistance of the participant. Food consumption and preparation steps may have also been missed between 30-s photo increments. Fruit, in particular, is often picked and consumed quickly, appearing in just 1 or 2 frames (out of approximately 1,500), or not at all. These issues may be addressed with an automated wearable camera with a higher frequency capture rate, although Arab & Winter (2010) reported that foods are still missed even when using an automated wearable camera with 10-s frequency of data capture [16], and a higher image capture frequency would also increase the number of images that need to be coded, posing an additional burden on the research team.

The automated wearable camera used in this study (iON SnapCam Lite) named the captured images by image number rather than timestamp. Although the timestamp was embedded in the file’s metadata, it was not possible to see at-a-glance the time the photograph was taken to determine, for example, the timeslot in which the activity occurred or to easily calculate elapsed time. Furthermore, the automated wearable camera tended to "lose" time over the course of the study (Each day the automated wearable camera’s clock became increasingly out-of-sync with real time.). Therefore, even the timestamp embedded in the image’s metadata was not reliable, although it could be used to determine activity duration.

The positioning of the automated wearable camera around the neckline of the mother may also have resulted in missed data related to food/beverage consumption and activities, especially as it pertains to childcare and child feeding. For example, there was no visual record of foods consumed by the child under the care of someone else, potentially nutrient-rich ingredients added to a recipe prepared by another member of the household, passive caregiving when the mother was not facing her child and socializing while simultaneously engaged in other activities such as washing clothes. Future studies should carefully consider the placement of the automated wearable camera given the study objectives.

Finally, some participants had difficulty interpreting first-person perspective photographs captured by the wearable camera. This is notable because the image-assisted recall method is designed to "trigger" the participant’s recall of activities, however the images provide no assistance to (or worse, may misdirect) a participant’s recall of events if they cannot interpret what they see in the photo. Although participants easily identified the photos of their children and themselves, a substantial proportion struggled to interpret the photos of themselves engaged in a task. This issue has never before been reported, and it’s unclear what, if any, predictive value a participant’s skill at identifying a single still "action" image may have for their overall image-assisted recall performance. To reduce the burden of the method for participants and researchers in future studies, it would be useful to determine the minimum number of photos and the type of photo (e.g., a well-chosen single image) that is effective for improving recall.

A thorough analysis of the validity of automated wearable cameras and the 24-h recall methods were reported separately [3, 59].

### Strengths

Scalability is an important decision when choosing research methods. This feasibility study was conducted in communities with free-living participants, which reflects the conditions in which research is typically carried out in rural low-income countries. Compared to previous automated wearable camera feasibility studies, which are commonly conducted with populations less than a hundred, this study was conducted with a relatively large number of participants. This study also reports two innovative and beneficial research design choices. First, participants in this study were not responsible for operating the devices, which, although increasing the burden on the research team, likely reduced lost data. Second, an orientation to the automated wearable camera-captured images was added at the start of the image-assisted recall to help participants with little previous experience viewing first-person photography to use the images to trigger their memories. Future studies should explore whether these practices are effective in reducing reporting error.

### Limitations

This feasibility study was nested within a rigorous validation study, which necessitated the concurrent administration of multiple methods (e.g., 15-hour direct observation, 24-hour recalls and interactive voice response). To reduce the burden imposed on participants, the methods used to assess their perceptions of the AWC and IAR were limited to a brief semi-structured questionnaire at the end of each AWC data collection day and a simple survey of closed-ended questions at the end of the 5-day data collection period. Future studies should incorporate more robust qualitative methods to assess "acceptability" of the AWC in rural LIC contexts. For example, that nearly all participants rated the automated wearable camera-based image-assisted recall method as good or very good and were willing to participate in an automated wearable camera-based image-assisted recall study in the future was a surprising result. In-depth qualitative investigation is needed to unpack this finding.

 Also due to the concurrent collection of data via multiple methods, the interaction between the study team and participants was more intense in this study than would occur in typical studies using the automated wearable camera. Consequently, lost data was possibly lower in this study than it would ordinarily be because an observer in the home would have more readily identified a malfunctioning automated wearable camera and alerted the research team for quick replacement. Acceptability-related study withdrawals may have been lower in this study because the observers also helped to monitor negative rumors so the research team could address them early. However, acceptability may have been adversely affected because of high participant burden due to multiple simultaneous methods.

This study was conducted in a single population and so the results cannot be generalized to other populations, even to other rural populations in Uganda. Participants in this study spent most of their time at home. The feasibility of an automated wearable camera may be lower for participants who work away from home. The feasibility of the image-assisted recall, which took approximately 1-2-hours to administer, may also be lower in periods when workloads are heavy, and participants are experiencing time constraints e.g., during the planting or harvest seasons.

 Although the lack of significant differences in the household characteristics among those included and excluded from the study indicates that neither selection bias nor self-selection bias diminished the internal validity of the results among those recruited, the possible effect of the automated wearable camera on recruitment was not investigated. Future studies should try to monitor the reasons why people refuse to participate. For example, to better understand the factors that had influenced recruitment rates, non-participants could be asked to provide the reason, as done by Cowburn et al. (2015) [6]. Research to assess the perceptions of study participants’ family and community members regarding the automated wearable camera is also needed, as is research to estimate the cost-effectiveness of the automated wearable camera-based image-assisted recall method versus traditional recall methods, accounting for all equipment costs (including but not limited to the automated wearable cameras) and researcher time required.

## Conclusions

In conclusion, this study showed that using an automated wearable camera in rural Eastern Uganda was feasible. The results inform future studies about investments to improve feasibility in these contexts, including the need for higher quality devices, more automated data management processes, and more in-depth sensitization of study participants, their families, and communities.

The most critical issues were those that resulted in substantial lost and/or unusable data, e.g., automated wearable camera malfunction, poor image quality, and poor device usability. Although these challenges may be alleviated by an investment in higher quality cameras, they are commonly reported in other automated wearable camera studies using top-of-the-line devices explicitly designed for the purpose of behavioral research data collection.

Coding the automated wearable camera images for analysis was also resource intensive, a factor that must be considered in weighing the trade-offs between different data collection methods. This challenge, too, is commonly reported in other automated wearable camera studies. Rough estimates calculated ex post facto for this study, indicate that automated wearable camera image coding took approximately five researcher hours per participant day, substantially more than previously reported. Although not insurmountable, innovations engaging stakeholders in a wide array of fields (e.g., computer science, engineering, and social science) are needed to improve hardware, software, and data analysis methods for the automated wearable camera-based image-assisted recall method to be scalable, regardless of country context.

More unique to rural, low-income country settings, and therefore warranting special consideration for future studies in these contexts, are the lack of exposure to first-person photos, insufficient experience operating a computer (e.g., to independently review automated wearable camera-captured images), and different socio-cultural norms compared to the contexts in which current automated wearable camera protocols have evolved. In this study, separate tools were used by the participant to review their photos (a tablet computer) and by the enumerator collect the recall data (a paper-based, unstructured instrument). The image-assisted recall data was transcribed verbatim in the field and coded into the designated categories ex post facto. To improve the quality of data obtained via automated wearable cameras, future image-assisted recall studies should use a structured data format to reduce automated wearable camera image coding time; electronically code the data in the field, as an output of the image review process, to eliminate ex post facto data entry; and, ideally, use computer-assisted personal interviews (CAPI) software to ensure completion and reduce errors. In high-income countries, automated wearable camera-based image-assisted recall researchers have used the SenseCam browser, (63)Similar applications are needed for rural low-income country contexts where the image-assisted recall is administered at the participant's home (likely without access to electricity) and participants lack the skills to operate a computer. For these challenges, in-depth formative work is needed to specifically design automated wearable camera-based image-assisted recall methods that work for these contexts.

 There is no comparable method for capturing such rich and diverse data simultaneously and prospectively, on dimensions the participant may not think to report, or for enabling analysis of new questions that emerge after the data has been collected. Further work to design automated wearable camera-based image-assisted recall protocols is needed, however, to overcome the challenges common to rural, low-income country contexts and reduce the burdens posed on both participants and researchers and data losses.

## Supplementary Information

**Additional file 1: Figure 1.**

**Additional file 2: Figure 2.**

**Additional file 3: Supplementary Table 1.** Characteristics of households and mothers included in and excluded from the study.

**Additional file 4: Supplementary Table 2.** Participants' rating of their experience with the automated wearable camera-based image-assisted recall method^a^ (*N*=184).

**Additional file 5: Supplementary Table 3.** **a.** Participants' most favourite method. **b.** Participants' most favourite method: AWC-IAR vs OBS / WFR. **c.** Participants' most favourite method: AWC-IAR vs 24HR. **d.** Participants' most favourite method: AWC-IAR vs MP-IVR.

**Additional file 6: Supplementary Table 4.** **a.** Participants' least favourite method. **b.** Participants' least favourite method: AWC-IAR vs OBS / WFR. **c.** Participants' least favourite method: AWC-IAR vs 24HR. **d.** Participants' least favourite method: AWC-IAR vs MP-IVR.

**Additional file 7: Supplementary Table 5.** Participant's ability to recognize selected image types during the first and second image-assisted recall orientation (*N*=184).

**Additional file 8: Supplementary Table 6.** Participant's ability to recognize selected image types during the first and second image-assisted recall orientation.

**Additional file 9: Supplementary Table 7.** Lost data due to AWC inoperability - with and without observer present.

## Data Availability

The data that support the findings of this study are openly available in (Dataverse) at (URL), reference number (reference number).
