# Supplementary figures and images for: Automated wearable cameras for improving recall of diet and time use in Uganda: a cross-sectional feasibility study
Source: Nutr J. 2023 Jan 12;22:7. doi: 10.1186/s12937-022-00828-3 (PMC9835269; doi:10.1186/s12937-022-00828-3)

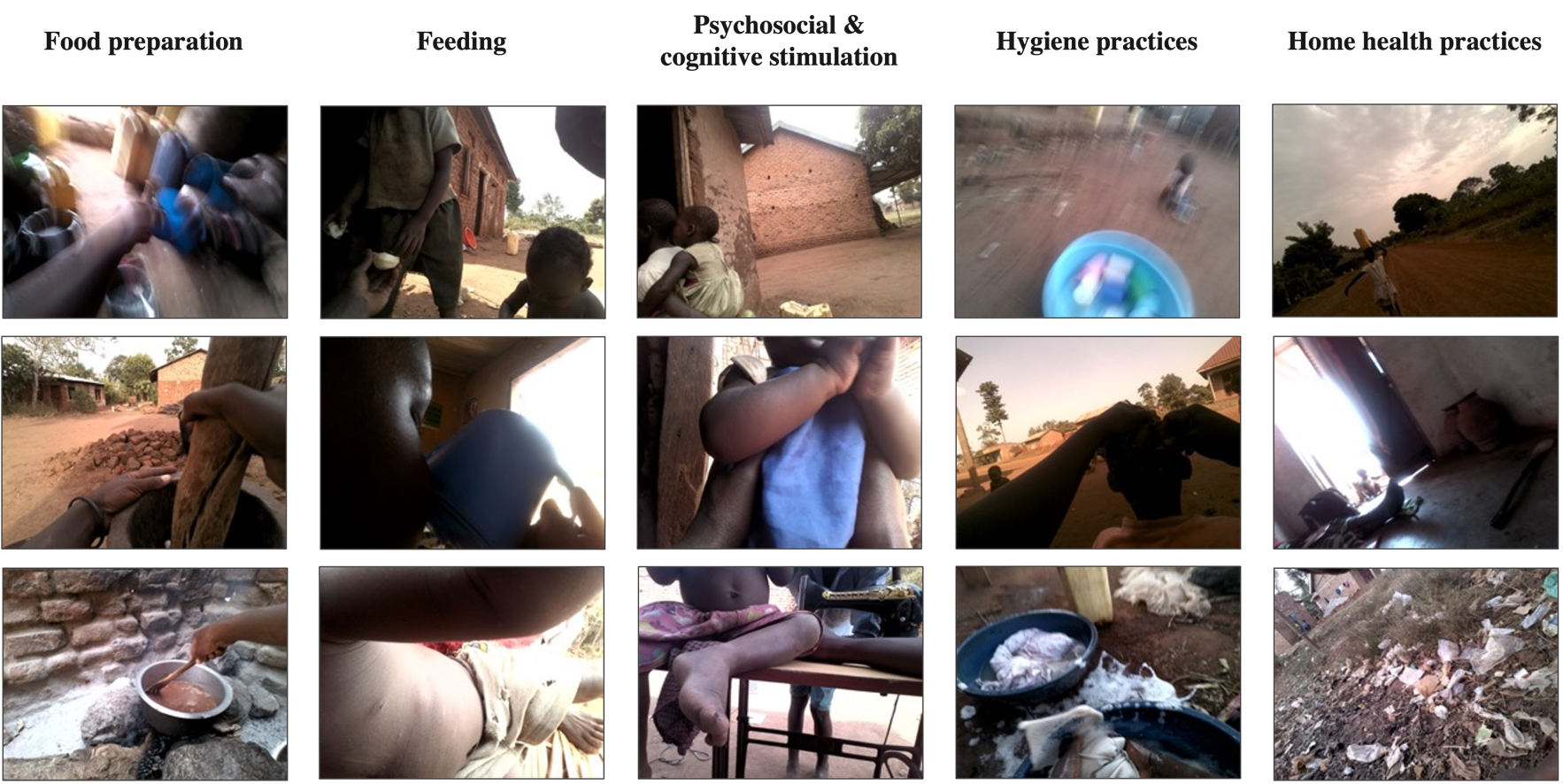

Supplement: Supplementary file 1 — Additional file 1: Figure 1. [file 12937_2022_828_MOESM1_ESM.png]

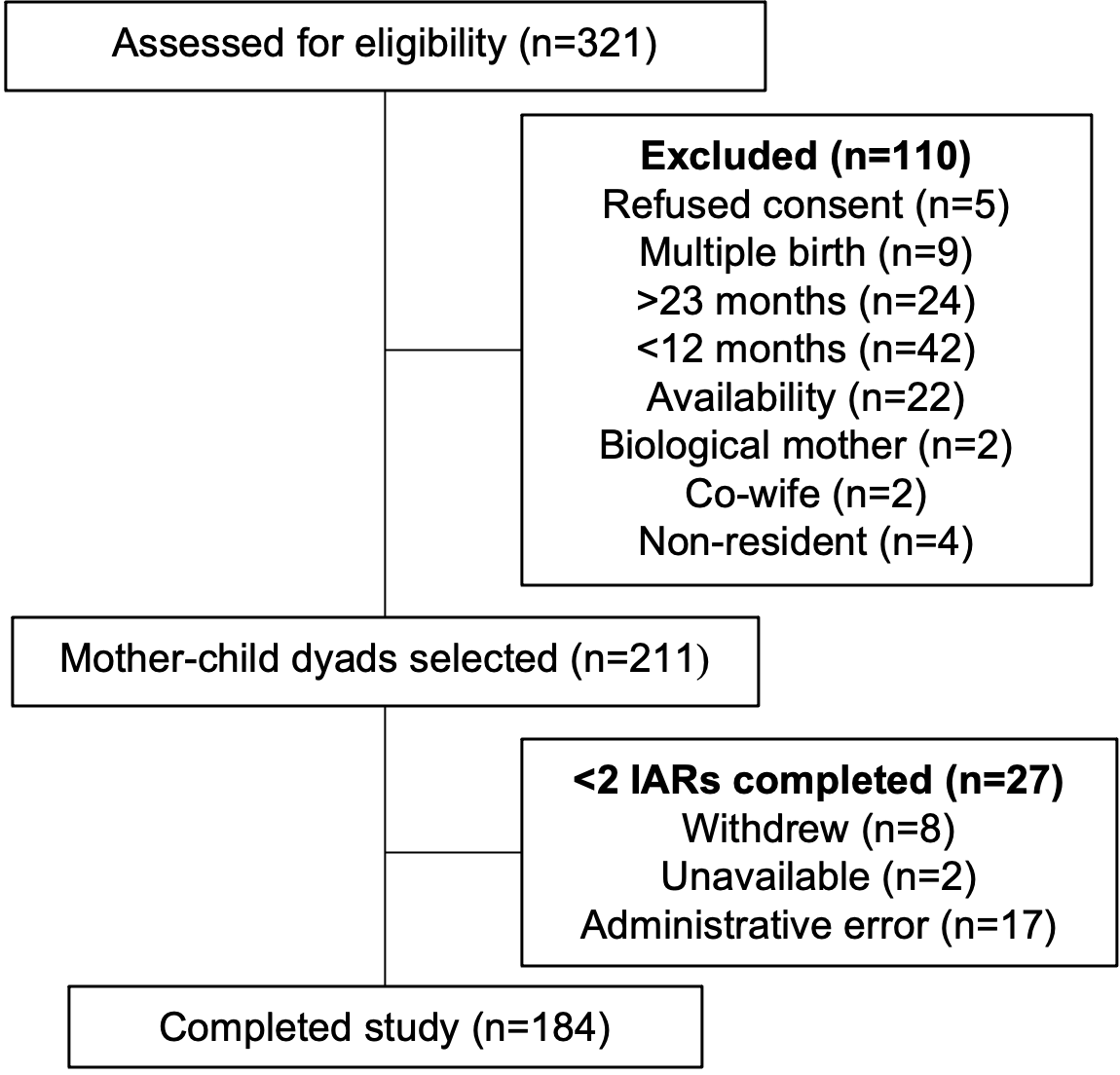

Supplement: Supplementary file 2 — Additional file 2: Figure 2. [file 12937_2022_828_MOESM2_ESM.png]
