# Supplementary material for: Automated wearable cameras for improving recall of diet and time use in Uganda: a cross-sectional feasibility study
Source: Nutr J. 2023 Jan 12;22:7. doi: 10.1186/s12937-022-00828-3 (PMC9835269; doi:10.1186/s12937-022-00828-3)
Supplement: Supplementary file 3 — Additional file 3: Supplementary Table 1. Characteristics of households and mothers included in and excluded from the study. [file 12937_2022_828_MOESM3_ESM.docx]

Supplementary Table 1. Characteristics of households and mothers included in and excluded from the study.

|  | Included | | |  | Excluded | | | | |  | *P* |
| --- | --- | --- | --- | --- | --- | --- | --- | --- | --- | --- | --- |
|  | n (%) | Median | 25^th^-75^th^ |  | n (%) | Median | | 25^th^-75^th^ | |  |  |
| Number of household members |  | 6.0 | 4.5, 8.0 |  |  | 7.0 | | 5.0, 8.5 | |  | .55 |
| Living below $1.25/day (2005 PPP) | 149 (20.1) |  |  |  | 14 (20.1) |  | |  | |  | .65 |
| Age (years) |  | 26 | 22, 30 |  |  | 23 | | 20, 29 | |  | .16 |
| Marital status  Married or living together  Divorced / Separated or Widowed  Never married and never lived together | 154 (85.6)  8 (4.4)  18 (10.0) |  |  |  | 23 (95.8)  0 (0.0)  1 (4.2) |  | |  | |  | .51 |
| Level of education - attended  None  Primary  Post-primary | 13 (7.1)  125 (67.9)  46 (25.0) |  |  |  | 1 (4.2)  17 (70.8)  6 (25.0) | |  | |  |  | 1.00 |
| Cannot read and write | 90 (50.0) |  |  |  | 12 (50.0) | |  | |  |  | 1.00 |
| Christian | 108 (58.7) |  |  |  | 15 (62.5) | |  | |  |  | .72 |
| PPP, purchasing power parity; P, p-value using Mann-Whitney U test to compare the medians and Fisher’s Exact test to compare the categorical data. | | | | | | | | | | | |
