# Supplementary material for: Automated wearable cameras for improving recall of diet and time use in Uganda: a cross-sectional feasibility study
Source: Nutr J. 2023 Jan 12;22:7. doi: 10.1186/s12937-022-00828-3 (PMC9835269; doi:10.1186/s12937-022-00828-3)
Supplement: Supplementary file 4 — Additional file 4: Supplementary Table 2. Participants' rating of their experience with the automated wearable camera-based image-assisted recall methoda (N=184). [file 12937_2022_828_MOESM4_ESM.docx]

Supplementary Table 2. Participants' rating of their experience with the automated wearable camera-based image-assisted recall method.^a^ (N=184)

| Rating | n (%) |
| --- | --- |
| Good or Very Good  Good  Very good | 170 (92.4)  67 (36.4)  103 (56.0) |
| Bad or Very bad  Bad  Very bad | 9 (4.9)  9 (4.9)  0 (0.0) |
| ^a^ How would you rate your experience with wearing the camera and looking at the photographs the next day? | |
