# Supplementary material for: Automated wearable cameras for improving recall of diet and time use in Uganda: a cross-sectional feasibility study
Source: Nutr J. 2023 Jan 12;22:7. doi: 10.1186/s12937-022-00828-3 (PMC9835269; doi:10.1186/s12937-022-00828-3)
Supplement: Supplementary file 5 — Additional file 5: Supplementary Table 3. a. Participants' most favourite method. b. Participants' most favourite method: AWC-IAR vs OBS / WFR. c. Participants' most favourite method: AWC-IAR vs 24HR. d. Participants' most favourite method: AWC-IAR vs MP-IVR. [file 12937_2022_828_MOESM5_ESM.docx]

Supplementary Table 3a. Participants' most favourite method.

|  | n (%) | P^1^ |
| --- | --- | --- |
| AWC-IAR | 44 (25.9) | <0.001 |
| OBS / WFR | 67 (39.4) |  |
| 24HR | 7 (4.1) |  |
| MP-IVR | 52 (30.6) |  |
| ^1^ P, p-value using the chi-square goodness-of-fit test to compare the categorical data | | |

Supplementary Table 3b. Participants' most favourite method: AWC-IAR vs OBS / WFR

|  | n (%) | P^1^ |
| --- | --- | --- |
| AWC-IAR | 44 (25.9) | 0.029 |
| OBS / WFR | 67 (39.4) |  |
| ^1^ P, p-value using the chi-square goodness-of-fit test to compare the categorical data.  * Indicates significant at 5% level, with the Bonferroni adjustment for 3 hypothesis (α=0.017). | | |

Supplementary Table 3c. Participants' most favourite method: AWC-IAR vs 24HR

|  | n (%) | P^1^ |
| --- | --- | --- |
| AWC-IAR | 44 (25.9) | <0.001* |
| 24HR | 7 (4.1) |  |
| ^1^ P, p-value using the chi-square goodness-of-fit test to compare the categorical data  * Indicates significant at 5% level, with the Bonferroni adjustment for 3 hypothesis (α=0.017). | | |

Supplementary Table 3d. Participants' most favourite method: AWC-IAR vs MP-IVR

|  | n (%) | P^1^ |
| --- | --- | --- |
| AWC-IAR | 44 (25.9) | 0.414 |
| MP-IVR | 52 (30.6) |  |
| ^1^ P, p-value using the chi-square goodness-of-fit test to compare the categorical data  * Indicates significant at 5% level, with the Bonferroni adjustment for 3 hypothesis (α=0.017). | | |
