# Supplementary material for: Automated wearable cameras for improving recall of diet and time use in Uganda: a cross-sectional feasibility study
Source: Nutr J. 2023 Jan 12;22:7. doi: 10.1186/s12937-022-00828-3 (PMC9835269; doi:10.1186/s12937-022-00828-3)
Supplement: Supplementary file 6 — Additional file 6: Supplementary Table 4. a. Participants' least favourite method. b. Participants' least favourite method: AWC-IAR vs OBS / WFR. c. Participants' least favourite method: AWC-IAR vs 24HR. d. Participants' least favourite method: AWC-IAR vs MP-IVR. [file 12937_2022_828_MOESM6_ESM.docx]

Supplementary Table 4a. Participants' least favourite method.

|  | n (%) | P^1^ |
| --- | --- | --- |
| AWC-IAR | 37 (28.5) | 0.041 |
| OBS / WFR | 31 (23.9) |  |
| 24HR | 42 (32.3) |  |
| MP-IVR | 20 (15.4) |  |
| ^1^ P, p-value using the chi-square goodness-of-fit test to compare the categorical data | | |

Supplementary Table 4b. Participants' least favourite method: AWC-IAR vs OBS / WFR

|  | n (%) | P^1^ |
| --- | --- | --- |
| AWC-IAR | 37 (28.5) | 0.467 |
| OBS / WFR | 31 (23.9) |  |
| ^1^ P, p-value using the chi-square goodness-of-fit test to compare the categorical data.  * Indicates significant at 5% level, with the Bonferroni adjustment for 3 hypothesis (α=0.017). | | |

Supplementary Table 4c. Participants' least favourite method: AWC-IAR vs 24HR

|  | n (%) | P^1^ |
| --- | --- | --- |
| AWC-IAR | 37 (28.5) | 0.574 |
| 24HR | 42 (32.3) |  |
| ^1^ P, p-value using the chi-square goodness-of-fit test to compare the categorical data  * Indicates significant at 5% level, with the Bonferroni adjustment for 3 hypothesis (α=0.017). | | |

Supplementary Table 4d. Participants' least favourite method: AWC-IAR vs MP-IVR

|  | n (%) | P^1^ |
| --- | --- | --- |
| AWC-IAR | 37 (28.5) | 0.024 |
| MP-IVR | 20 (15.4) |  |
| ^1^ P, p-value using the chi-square goodness-of-fit test to compare the categorical data  * Indicates significant at 5% level, with the Bonferroni adjustment for 3 hypothesis (α=0.017). | | |
