# Supplementary material for: Automated wearable cameras for improving recall of diet and time use in Uganda: a cross-sectional feasibility study
Source: Nutr J. 2023 Jan 12;22:7. doi: 10.1186/s12937-022-00828-3 (PMC9835269; doi:10.1186/s12937-022-00828-3)
Supplement: Supplementary file 7 — Additional file 7: Supplementary Table 5. Participant's ability to recognize selected image types during the first and second image-assisted recall orientation (N=184). [file 12937_2022_828_MOESM7_ESM.docx]

Supplementary Table 5. Insufficient number of images due to AWC inoperability, by week

| Issue |  | Participants Affected by Week | | | | | | | |
| --- | --- | --- | --- | --- | --- | --- | --- | --- | --- |
|  | Total  (N=184) | Bugiri (N=142) | | | | | Kamuli (N=69) | | |
|  |  | *WK1* | *WK2* | *WK3* | *WK4* | *LD Total* | *WK5* | *WK6* | *LD Total* |
|  | *n (%)* | *n* | *n* | *n* | *n* | *n (%)* | *n* | *n* | *n (%)* |
| Participants with insufficient number of images due to AWC inoperability* | 27 (14.7) | 0 | 4 | 1 | 4 | 9 (6.3) | 7 | 11 | 18 (26.1) |
| * Defined as 12 hours of images (1,440) per 15-hour day (1,800).  WK, week; IAR, image-assisted recall. | | | | | | | | | |
