# Supplementary material for: Automated wearable cameras for improving recall of diet and time use in Uganda: a cross-sectional feasibility study
Source: Nutr J. 2023 Jan 12;22:7. doi: 10.1186/s12937-022-00828-3 (PMC9835269; doi:10.1186/s12937-022-00828-3)
Supplement: Supplementary file 8 — Additional file 8: Supplementary Table 6. Participant's ability to recognize selected image types during the first and second image-assisted recall orientation. [file 12937_2022_828_MOESM8_ESM.docx]

Supplementary Table 6. Participant's ability to recognize selected image types during the first and second image-assisted recall orientation.

| Orientation photo type | IAR1 | IAR2 | *P** |
| --- | --- | --- | --- |
|  | n (%) | n (%) |  |
| **Photo of herself**  Recognized  Did not recognize | 171 (98.8)  2 (1.2) | 172 (97.7)  4 (2.3) | 0.688 |
| **Photo of her child**  Recognized  Did not recognize | 184 (100.0)  0 (0.0) | 180 (98.9)  2 (1.1) | 0.500 |
| **Photo of her home**  Recognized  Did not recognize | 180 (97.8)  4 (2.2) | 178 (97.3)  5 (2.7) | 1.000 |
| **Photo of her garden**  Recognized  Did not recognize | 136 (93.2)  10 (6.9) | 142 (96.6)  5 (3.4) | 0.039 |
| **Photo of her own hand while performing a task**  Recognized  Did not recognize | 164 (89.1)  20 (10.9) | 175 (95.6)  8 (4.4) | 0.036 |
| P, p-value using McNemar's exact test to compare the paired categorical data. | | | |
