# Supplementary material for: Automated wearable cameras for improving recall of diet and time use in Uganda: a cross-sectional feasibility study
Source: Nutr J. 2023 Jan 12;22:7. doi: 10.1186/s12937-022-00828-3 (PMC9835269; doi:10.1186/s12937-022-00828-3)
Supplement: Supplementary file 9 — Additional file 9: Supplementary Table 7. Lost data due to AWC inoperability - with and without observer present. [file 12937_2022_828_MOESM9_ESM.docx]

Supplementary Table 7. Lost data due to AWC inoperability - with and without observer present

|  | Lost data due to inoperability | |
| --- | --- | --- |
|  | Observer present | Observer NOT present |
| AWC-IAR1 (n=184) | 8 | 20 |
| AWC-IAR2 (n=184) | 16 | 12 |
| Total | 24 (6.5) | 32 (8.7) |
